# Epidemiological Society. Quarterly Report

**Published:** 1855-12

**Authors:** 


### EPIDEMIOLOGICAL SOCIETY. QUARTERLY REPORT.

On Monday, November 5th, 1855, Dr. Babington, the President, having in a few preliminary remarks taken a rapid glance at the main proceedings of the Society during the past year, Mr. Grainger inaugurated this, the sixth session of the Society, by an address (which appears in our present number,) on the subject of "The Noxious Influence of Animal Effluvia, with Observations on some of the principles of Sanitary Science."

On Monday, December 3rd, a paper on "Puerperal Fever," was read by Professor Murphy. (A second paper on the same subject will be read by the Professor, most probably in the course of the present session.)

The following donations have been received during the quarter.

From John M'Lennan, M.D., late Physician General, Bombay army, and President of the Medical Board, the sum of £5, in aid of the funds of the Society.—Transactions of the Medical and Physical Society of Bombay. Presented by the Society.—" Report on the Cholera Outbreak in the parish of St. James's, Westminster." Presented by the Committee appointed by the Vestry.

The following gentlemen have become members of the Society. *As Resident Members.*—Edward Wm. Murphy, M.D., Professor of Midwifery, University College, London; James Honeywood, M.R.C.S., 54, Nelson Square. *As Non-resident Members.*—H. W. Rumsey, F.R.C.S., Cheltenham; R. S. Stedman, M.R.C.S., Sharnbrook, Beds; T. E. Jacobson, Esq., Sleaford. *As Corresponding Member.*—John Hancock, M.R.C.S., etc., Wedmore, Somerset.

The Society has to deplore the loss, by death, during the quarter, of two valued members of the society and of the profession. 1. H. F. Gough, M.D., late Physician General, Bengal. Dr. Gough, after a long life, passed with honour and distinction in India, returned to this country, and became a member and one of the vice-presidents of the Society. Dr. Gough manifested his zeal for the prosperity of the Society by handsome donations to its funds. 2. George Pilcher, F.R.C.S., who was suddenly removed from amongst us in the prime of life and intellectual vigour. Mr. Pilcher was a member of council from the foundation of the Society up to the period of his death, and took great interest in the Society, and in sanitary questions generally.

---

During the last nine months, the *Transactions of the Epidemiological Society* have been presented to the public in the pages of "The Quarterly Journal of Public Health," edited by Dr. Richardson.

In return for the supply of the proceedings of the Society, Dr. Richardson has kindly arranged, that the "Transactions" are published in his Journal so as to form a separate department, which, at the end of the year, can be removed and bound in a distinct volume, without injury to the other parts of the Journal.

This public-spirited liberality is duly appreciated by the Council of the Epidemiological Society; and it will be gratifying to them to learn, that the appearance of the "Transactions" in the pages of the Journal of Public Health have in any degree contributed to its more extensive circulation.

---

RICHARDS, 37, GREAT QUEEN STREET.

