# Erratum

**Published:** 1856-04

**Authors:** 


### ERRATUM.

In our last number, when deploring the loss the Society had suffered by the death of one of its Vice-Presidents, Dr. H. F. Hough, the name was inadvertently printed *Gough*.

